# Differentiation of Lung Malignancy from Benign Lesions in Patients with Nontuberculous Mycobacterial Infection: A Retrospective Analysis of Biopsy-Proven Cases

**DOI:** 10.3390/diagnostics16091321

**Published:** 2026-04-28

**Authors:** Wonju Hong, In Jae Lee, Lyo Min Kwon, Min-Jeong Kim, Hyunseung Nam

**Affiliations:** 1 Department of Radiology, Hallym University Sacred Heart Hospital, College of Medicine, Hallym University, Anyang 14068, Gyeonggi-do, Republic of Korea; 2 Department of Critical Care Medicine, Hallym University Sacred Heart Hospital, College of Medicine, Hallym University, Anyang 14068, Gyeonggi-do, Republic of Korea

**Keywords:** nontuberculous mycobacteria, nontuberculous mycobacterial pulmonary disease, tomography, X-ray computed, lung neoplasms, percutaneous core needle biopsy, radiologic surveillance

## Abstract

**Background/Objectives:** Nontuberculous mycobacterial (NTM) pulmonary infection may present with diverse radiologic manifestations that mimic lung malignancy. Furthermore, in patients with newly detected or progressively enlarging pulmonary lesions, distinguishing benign NTM-related lesions from coexisting malignancy is often challenging. When imaging findings are indeterminate, percutaneous core needle biopsy (PCNB) may be required for diagnostic clarification. This study aimed to evaluate the pathologic results, clinical characteristics, and CT features of pulmonary lesions in patients with NTM infection who underwent PCNB and to identify factors associated with malignancy. **Methods:** This retrospective study included 38 patients with NTM infection who underwent CT-guided PCNB for lung lesions between 2015 and 2025. Two blinded radiologists reviewed chest CT scans obtained within six weeks prior to the biopsies. Clinical variables and CT features were compared between malignant and benign lesions. Univariable and multivariable logistic regression analyses were performed to explore factors associated with malignancy. **Results:** Of the 38 patients, 9 (23.7%) were diagnosed with malignancy and 29 (76.3%) had benign lesions. Malignant lesions more frequently demonstrated a lobulated irregular morphology compared to benign lesions (88.9% vs. 37.9%). Emphysema was also more common in the malignancy group (88.9% vs. 24.1%, *p* < 0.001). In the multivariable logistic regression analysis, lobulated irregular morphology (odds ratio [OR], 19.856; 95% CI, 1.516–260.089; *p* = 0.023) and emphysema (OR, 35.531; 95% CI, 2.857–441.824; *p* = 0.005) were associated with malignancy. However, the wide confidence intervals indicate substantial uncertainty due to the limited number of malignant cases. **Conclusions:** In patients with NTM infection who underwent PCNB for suspicious lung lesions, a lobulated irregular morphology and the presence of emphysema were associated with malignancy in this exploratory cohort. These findings may provide useful information to support clinical decision-making regarding biopsy in patients with NTM infection and indeterminate pulmonary lesions.

## 1. Introduction

Nontuberculous mycobacteria (NTM) are environmental bacteria that cause opportunistic infection in humans [1,2]. Pulmonary disease is the predominant clinical manifestation [1], and NTM pulmonary disease is an increasingly recognized chronic infection with rising incidence and prevalence worldwide. In South Korea, the annual prevalence increased from 5.3 to 41.7 cases per 100,000 population between 2008 and 2018 [3,4].

The diagnosis of NTM pulmonary disease is complex and requires a combination of clinical, microbiological, and radiologic criteria established by the American Thoracic Society and the Infectious Disease Society of America (ATS/IDSA) [5]. Chest computed tomography (CT) plays a pivotal role in the radiologic evaluation of NTM pulmonary disease. Although the two major radiologic patterns of NTM pulmonary disease, the fibrocavitary form and the nodular bronchiectatic form, are well recognized, imaging findings alone are often insufficient for a definitive diagnosis [6,7]. Importantly, NTM-related pulmonary lesions may present as solitary nodules, masses, or consolidations that closely mimic lung cancer [7,8]. Prior studies have shown that pulmonary nodules caused by NTM are often difficult to distinguish from malignancy during preoperative evaluations, frequently necessitating a tissue diagnosis in clinically indeterminate cases [7,8,9]. In addition, chronic NTM infection has been suggested to increase the risk of lung cancer; the coexistence of NTM infection and pulmonary malignancy is not uncommon, further complicating radiologic interpretation and clinical decision-making [10]. Distinguishing benign NTM-related lesions from a concurrent malignancy is often challenging in clinical practice, particularly when newly detected, persistent, or enlarging lesions are identified on follow-up CT scans. In such cases, percutaneous core needle biopsy (PCNB) is often required to obtain a definitive histopathologic diagnosis [6,11].

Data remain limited regarding which clinical and CT features help distinguish benign NTM-related lesions from coexisting malignancy, specifically among patients with NTM infection who undergo biopsy for suspicious pulmonary lesions. We hypothesized that specific clinical factors and CT features would be associated with malignancy in this setting. Identifying such features could improve risk stratification, support the timely biopsy of lesions that are more likely to represent lung cancer, and potentially reduce unnecessary invasive procedures for benign disease. Therefore, this study aimed to evaluate the pathologic results, clinical characteristics, and CT features of pulmonary lesions in patients with NTM infection who underwent PCNB and to identify clinical and radiologic predictors of malignancy.

## 2. Materials and Methods

### 2.1. Study Population

This retrospective study was approved by the institutional review board of Hallym University Sacred Heart Hospital and informed consent was waived.

**Figure 1:**
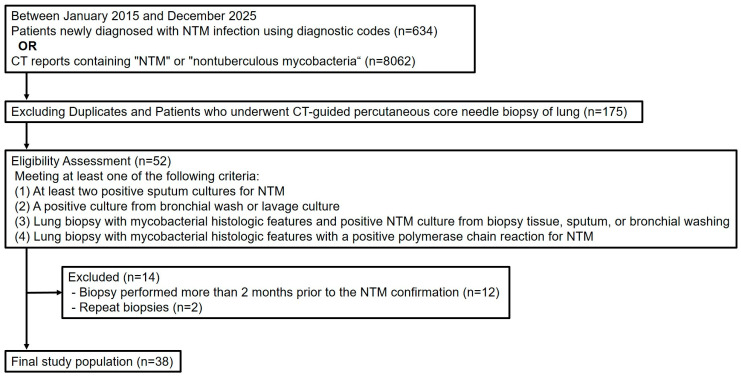
Flowchart of the study population.

Between January 2015 and December 2025, we identified patients newly diagnosed with NTM infection at our institution using diagnostic codes. During the same period, we also retrieved chest CT examinations whose radiology reports contained the terms “NTM” or “nontuberculous mycobacteria” using a radiology report search program. We used both search methods to reduce the likelihood of missing potentially eligible patients who may not have been captured by the initial diagnostic code search. From these initial search results, we included patients who had undergone CT-guided PCNB for lung lesions. After a comprehensive review of the electronic medical records (EMRs), patients were eligible for inclusion if they met at least one of the following microbiological or histological criteria: (1) at least two positive sputum cultures for NTM, (2) a positive culture from bronchial wash or lavage fluid, (3) a lung biopsy with mycobacterial histologic features and positive NTM culture from biopsy tissue, sputum, or bronchial washing, or (4) a lung biopsy showing mycobacterial histologic features with a positive polymerase chain reaction (PCR) test for NTM. Patients meeting at least one of these criteria were considered to have NTM infection. After excluding duplicate data, an initial cohort of 52 patients who underwent PCNB was identified. We excluded biopsies performed more than two months prior to the microbiologic or histologic identification of mycobacterial infection. In cases in which repeat biopsies were performed on the same lesion, only the initial biopsy was included. Ultimately, a total of 38 patients were included in the final analysis (Figure 1).

For the final study population, the following variables were recorded from the EMRs: age, sex, presence of an underlying malignancy, date of NTM infection diagnosis, date of PCNB, and chest CT scans performed before and after the PCNB. All included patients had undergone a chest CT within six weeks prior to the PCNB procedure.

Initially, 634 patients were identified via diagnostic codes, and 8062 CT examinations were retrieved from radiology reports. Of these, 175 patients underwent CT-guided percutaneous core needle biopsy. After applying eligibility criteria and removing 16 duplicate cases, 52 patients remained. An additional 14 patients were excluded: 12 whose biopsies were performed more than two months prior to microbiologic or histologic confirmation, and 2 with repeat biopsies. Ultimately, 38 patients were included in the final analysis.

### 2.2. Microbiologic Studies

Respiratory specimens, including sputum, bronchial washings, lavage fluid, aspirates, or tissue obtained from PCNB, were used for the microbiologic diagnosis of NTM infection. Mycobacterial smears and cultures were performed using standard methods [12]. PCR assays targeting *Mycobacterium tuberculosis* and NTM were also conducted on the specimens, and specific NTM species were identified.

### 2.3. Histopathology

The pathologic reports of the PCNB specimens were reviewed. In cases of chronic granulomatous inflammation with or without necrosis, Ziehl-Neelsen staining was performed. Real-time PCR for *Mycobacterium tuberculosis* (TB-PCR) and nontuberculous mycobacteria (NTM-PCR) was conducted on tissue samples when requested by the clinician or deemed appropriate by a pathologist. The PCR results were reported separately, but in conjunction with the histopathological findings.

Lesions were classified as malignant when the pathologic findings demonstrated a specific malignancy. Lesions were classified as benign when the pathologic findings were consistent with mycobacterial histologic features accompanied by positive culture or PCR results from the specimen, or when the lesion remained stable or decreased in size on follow-up CT for at least two years.

### 2.4. Imaging Techniques and Analyses of CT Features

CT examinations performed prior to PCNB were acquired using one of the following scanners: Brilliance 64 (Philips Medical Systems, Best, The Netherlands), SOMATOM Definition Flash, or SOMATOM Definition Edge (Siemens Healthineers, Erlangen, Germany), Aquilion (Canon Medical Systems, Otawara, Japan), or iCT 256 (Philips Medical Systems, Best, The Netherlands). Twenty-seven patients underwent contrast-enhanced CT, including non-contrast and contrast phases, using automatic exposure control. The contrast-enhanced phase was initiated 50 s after the intravenous administration of 80–100 mL of contrast agent at a rate of 2 mL/s. In eleven patients, noncontrast chest CT scans were performed. Images were reconstructed using a lung reconstruction algorithm with 3 mm and/or 1 mm slice thicknesses.

CT features were analyzed by two thoracic radiologists (with 28 and 6 years of clinical experience, respectively), both of whom were blinded to the pathologic reports and final diagnoses. In cases of discrepancy, a consensus was reached through discussion.

The following CT characteristics of the targeted lesions were evaluated: location (lung lobe); morphologic appearance (lobulated irregular nodule, smooth round nodule, patchy consolidation, or subpleural thickening); maximum diameter; presence of cavitation; presence of low attenuation (<20 Hounsfield Unit on a non-contrast scan) within the solid component suggestive of necrosis or fluid; presence of associated multiple nodules; presence of the two typical radiologic forms of NTM pulmonary disease (nodular bronchiectatic or upper lobe fibrocavitary) in the underlying lung parenchyma; emphysema; lymphadenopathy; and pleural effusion. If CT scans obtained prior to the examination immediately preceding the PCNB were available, interval changes in the biopsied lesion as well as in other pulmonary lesions were also assessed.

### 2.5. Procedure of Percutaneous Core Needle Biopsy

All PCNB procedures were performed under CT guidance using one of the following scanners: Brilliance 64 (Philips Medical Systems, Best, The Netherlands), Sensation 64, or SOMATOM Definition Edge (Siemens Healthineers, Erlangen, Germany). Biopsies were performed using an automated biopsy gun with an 18-G needle (Magnum, Bard, NJ, USA). A 1.5 or 2.2 cm stroke length was selected based on the size of the target lesion.

Prior to biopsy, a preprocedural CT scan was obtained to determine the most optimal and safest needle trajectory for the target lesion. Patient positioning (supine, prone, or oblique) was selected based on the location of the pulmonary lesion and the intended puncture site. When indicated, additional samples for microbiological culture were aspirated using a coaxial introducer after core needle biopsy. Immediately after the procedure, a postprocedural CT scan was performed to evaluate procedure-related complications. All procedures were performed by one of three experienced thoracic radiologists.

### 2.6. Statistical Analyses

Because of the retrospective design, no formal sample size calculation was performed, and all eligible patients identified during the study period were included in the analysis. All statistical analyses were performed using SPSS version 30.0 (IBM Corp., Armonk, NY, USA). Continuous data were compared using the independent *t*-test or Mann–Whitney *U* tests, as appropriate, based on the normality of the distribution. Categorical data were compared using Pearson’s chi-squared test or Fisher’s exact test. Interobserver agreement between the two readers for CT findings was assessed using unweighted Cohen’s kappa statistic for nominal categorical variables, with 95% confidence intervals.

To identify factors associated with malignancy, univariable logistic regression analysis was performed. Variables with a *p*-value < 0.10 in the univariable analysis were included in the multivariable logistic regression analysis using a backward stepwise conditional method to account for potential confounding. A *p*-value < 0.05 was considered to be statistically significant.

## 3. Results

### 3.1. Clinical Characteristics, CT Features, and Pathologic Results of PCNB

A total of 38 patients with microbiologic or histologic evidence of NTM infection who underwent PCNB for pulmonary lesions were included in the study. The patients were divided into two descriptive groups based on biopsy timing relative to their initial NTM diagnosis, as this reflected two distinct clinical scenarios with potentially different indications for biopsy. Group 1 included patients who underwent PCNB during follow-up or surveillance after an established diagnosis of NTM infection, usually because of newly developed or interval-changing pulmonary lesions. Group 2 included patients who underwent PCNB at or near the time of initial diagnosis (within one month), where tissue diagnosis aided the concurrent evaluation of NTM infection and possible malignancy.

The baseline characteristics and pathologic results are summarized in Table 1, and chest CT findings are summarized in Table 2.

Group 1 consisted of 22 patients. The mean age of this group was 70.3 ± 7.4 years, with a male predominance (72.7%, 16/22). The median interval between the diagnosis of NTM infection and PCNB was 551.5 days. The most frequently isolated NTM species was *Mycobacterium avium* (59.1%, 13/22), followed by *Mycobacterium intracellulare* (18.2%, 4/22). Underlying malignancies were present in eight patients (36.4%), including colon, bladder, gallbladder, and nasopharyngeal cancers.

Group 2 comprised 16 patients. The mean age was 67.4 ± 11.2 years, with a slightly higher proportion of females (56.3%, 9/16). The median interval between diagnosis and biopsy was 6.5 days. *Mycobacterium avium* and *Mycobacterium intracellulare* were equally isolated, each accounting for 37.5% of the cases. Two patients (12.5%) had a history of underlying malignancy (one breast cancer and one concurrent hypopharynx, esophageal, and colon cancer).

Pathologically, malignancy was confirmed in 7 of the 22 patients (31.8%) in Group 1, with squamous cell carcinoma being the most common subtype. In contrast, 2 of the 16 patients (12.5%) in Group 2 were diagnosed with malignancy. The majority of biopsies in both groups yielded benign findings (15 in Group 1 and 14 in Group 2), predominantly exhibiting chronic inflammation with or without necrosis.

With respect to CT findings, lobulated irregular nodules were the most common morphologic pattern in both groups (50.0% [11/22] in Group 1 and 50.0% [8/16] in Group 2), followed by smooth round nodules and patchy consolidations. Associated multiple nodules were common in both groups (72.7% in Group 1 and 87.5% in Group 2). Notably, while the nodular bronchiectatic form was frequently observed in the underlying lung parenchyma of Group 1 (54.5%, 12/22), the majority of Group 2 patients (68.8%, 11/16) did not exhibit either of the typical radiological forms of NTM pulmonary disease. Emphysema was present in 50.0% (11/22) of Group 1 and 25.0% (4/16) of Group 2, respectively. However, the wide confidence intervals reflect the limited sample size and should be interpreted cautiously. Interobserver agreement for CT findings was generally good to excellent, with unweighted Cohen’s kappa values ranging from 0.707 to 1.000. Detailed agreement statistics are presented in Supplementary Table S1.

### 3.2. Clinical and Radiologic Characteristics of Benign and Malignant PCNB Lesions

Of the 38 patients analyzed across the entire cohort, 9 patients (23.7%) were diagnosed with malignancy, while 29 patients (76.3%) had benign lesions (Table 3). The mean age of patients in the malignancy group was significantly higher than that in the benign group (74.7 ± 8.6 vs. 67.3 ± 9.1 years; *p* = 0.045). There was no significant difference in sex distribution between the two groups (*p* = 0.061). Emphysema was significantly more prevalent in the malignancy group compared to the benign group (88.9% vs. 24.1%, *p* < 0.001). Other parameters, including lesion size, location, presence of cavity or necrosis, lymphadenopathy, pleural effusion, underlying typical radiologic forms of NTM pulmonary disease, and interval changes of the PCNB lesions or other lung lesions, did not differ significantly between the two groups.

**Figure 2:**
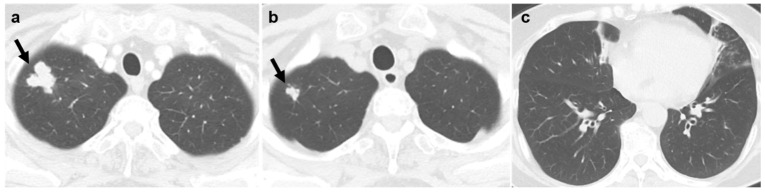
A female patient with nontuberculous mycobacterial infection. (**a**) Chest CT demonstrates a 2.8 cm lobulated irregular nodule (arrow) in the right upper lobe, confirmed as squamous cell carcinoma by percutaneous core needle biopsy. (**b**) On CT scan obtained 18 months earlier, the nodule measured 1.2 cm (arrow). (**c**) Mild bronchiectasis and nodular infiltration are observed in the right middle lobe and the lingular segment of the left upper lobe.

**Figure 3:**
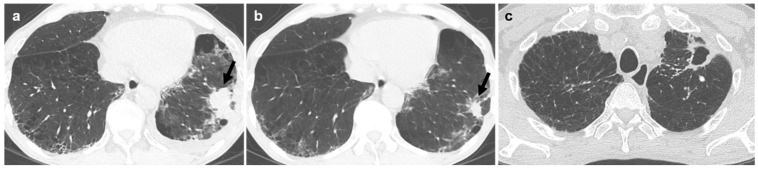
A male patient with nontuberculous mycobacterial infection. (**a**) Chest CT demonstrates a 3.6 cm lobulated irregular nodule (arrow) in the left lower lobe. Poorly differentiated carcinoma was diagnosed via percutaneous core needle biopsy. (**b**) On CT scan obtained 6 months earlier, the lesion measured 1.7 cm (arrow). Emphysema was present in underlying lung. (**c**) A fibrocavitary pattern of nontuberculous mycobacterial pulmonary disease is present in the left upper lobe.

**Figure 4:**
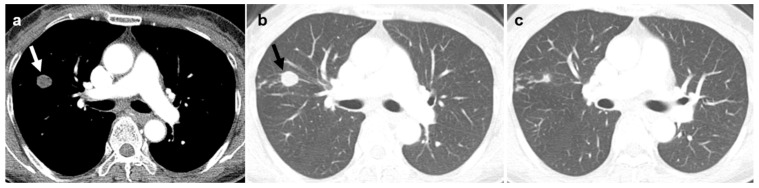
A female patient with nontuberculous mycobacterial infection. (**a**,**b**) Chest CT reveals a 1.6 cm round nodule (arrow) with internal low attenuation in the right upper lobe. Percutaneous core needle lung biopsy indicated chronic inflammation with extensive necrosis, and tissue PCR results were positive. (**c**) Adjacent associated small nodular infiltrations are present in the right upper lobe.

**Figure 5:**
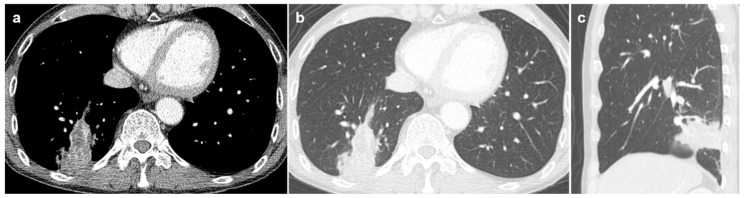
A male patient with nontuberculous mycobacterial infection. (**a**–**c**) Chest CT shows a focal patchy consolidation with internal low attenuation in right lower lobe, which had persisted for two months. Percutaneous core needle biopsy of the lesion revealed chronic granulomatous inflammation with necrosis. Subsequent bronchial lavage fluid culture was positive for NTM.

Regarding morphologic appearance, lobulated irregular nodules were significantly more prevalent in malignant lesions compared to benign lesions (88.9% vs. 37.9%; Figure 2 and Figure 3). In contrast, benign lesions manifested as smooth round nodules, patchy consolidations, or subpleural thickening more frequently than malignant ones (Figure 4 and Figure 5). Although malignant lesions were less frequently associated with multiple nodules (55.6% vs. 86.2%), this difference did not reach statistical significance (*p* = 0.071).

In the univariable logistic regression analysis, older age (odds ratio [OR], 1.106; 95% confidence interval [CI], 1.000–1.222; *p* = 0.049), lobulated irregular nodule shape (OR, 13.091; 95% CI, 1.436–119.338; *p* = 0.023), and the presence of emphysema (OR, 25.143; 95% CI 2.660–237.624; *p* = 0.005) were associated with malignancy. In the multivariable logistic regression analysis, a lobulated irregular morphology (OR, 19.856; 95% CI, 1.516–260.089; *p* = 0.023) and emphysema (OR, 35.531; 95% CI, 2.857–441.824; *p* = 0.005) remained associated with malignancy. The wide confidence intervals reflect statistical uncertainty related to the limited number of malignant cases.

## 4. Discussion

This study investigated the clinical and radiologic characteristics of pulmonary lesions in patients with NTM infection who underwent CT-guided PCNB and explored features associated with malignancy. Among 38 patients with indeterminate lesions requiring tissue diagnosis, malignancy was identified in nine patients (23.7%). In our cohort, lobulated irregular morphology and the presence of emphysema were associated with malignancy on multivariable analysis. However, due to the exploratory nature of the study, the limited sample size, the small number of malignant cases, and the wide confidence intervals, these findings should be interpreted cautiously.

The prevalence of malignancy in our cohort (23.7%, 9/38) was higher than the 2.0% to 8.6% reported in general NTM pulmonary disease surveillance studies [10,13,14,15]. This discrepancy likely reflects the highly selected nature of our study population, which consisted only of patients with clinically or radiologically indeterminate pulmonary lesions who underwent biopsy. Although biopsy for suspicious lesions is frequently required in clinical practice, data regarding factors associated with malignancy in this specific setting remain limited. In addition, our study included two patients who did not fully meet the ATS/IDSA microbiologic criteria but had compatible biopsy findings and positive NTM-PCR results, reflecting the increasing use of molecular diagnostics in clinical practice. This approach was intended to reflect real-world diagnostic practice. More importantly, our findings support the concept that pulmonary malignancy may coexist with NTM infection and complicate the interpretation of suspicious pulmonary lesions.

A notable finding of this study was the association between a lobulated irregular morphology and malignancy. In contrast, benign lesions more frequently appeared as smooth round nodules or patchy consolidations. These findings are consistent with previous studies demonstrating malignant lesions often demonstrate irregular, lobulated, or spiculated morphology [10,13], whereas NTM infection can mimic cancer with solitary nodules or focal consolidations [7,15,16,17,18]. This suggests that lesion morphology remains an important component of radiologic risk assessment even in patients with microbiologic or histologic evidence of NTM infection.

Emphysema was also highly prevalent in the malignancy group (88.9% vs. 24.1%) in our analysis. This finding should be interpreted carefully, however, as it may reflect underlying smoking exposure, chronic obstructive pulmonary disease (COPD), or an increased baseline risk of lung cancer rather than serving as a specific imaging feature distinguishing malignant from benign lesions [19,20]. Furthermore, emphysematous lungs may be more susceptible to NTM colonization and infection due to impaired mucociliary clearance [21]. Because detailed smoking history and COPD-related clinical data were not fully available for adjustment in the present study, residual confounding cannot be excluded. Accordingly, this finding should be regarded as exploratory and hypothesis-generating.

Other radiologic features, including lesion size, cavitation, associated nodules, and interval changes, were not significantly different between benign and malignant groups. The overlapping presentation of cavitation in both NTM infection and squamous cell carcinoma may limit its utility as a reliable discriminator. Although malignant lesions tended to be less frequently associated with multiple nodules, this finding did not reach statistical significance. Similarly, older age was associated with malignancy in univariable analysis, but not after multivariable adjustment. These findings underscore the difficulty of relying on any single clinical or imaging feature when evaluating suspicious pulmonary lesions in this population.

The clinical relevance of this issue may be particularly important in Asian populations, where mycobacterial lung diseases, including both tuberculosis and NTM infection, remain important considerations in everyday practice. In such settings, the differential diagnosis between infectious or inflammatory pulmonary lesions and lung malignancy can be especially challenging. This issue may become even more important as CT-based detection of pulmonary nodules increases and as lung cancer in never-smokers receives greater attention in Asia [22]. Prior screening research from Taiwan suggested that conventional smoking-based eligibility criteria fail to identify a substantial portion of lung cancer, particularly among women and never-smokers [22]. In this context, accurate radiologic assessment and appropriate selection for tissue diagnosis are particularly important.

In our study, a subset of patients (Group 2) underwent PCNB at or near the time of initial diagnosis because their imaging findings were atypical for NTM pulmonary disease and raised concern for possible malignancy. These cases illustrate the lung cancer-mimicking aspect of NTM infection [7]. In such situations, imaging findings and microbiologic results alone may be insufficient to exclude concurrent malignancy, and tissue diagnosis may be necessary to avoid delayed cancer treatment or unnecessary NTM therapy.

From a practical perspective, our findings may provide supportive information on whether biopsy should be considered in patients with NTM infection and indeterminate pulmonary lesions. Lesions with lobulated irregular morphology, particularly in the presence of emphysema, may raise concern for malignancy. Conversely, lesions with less suspicious morphology and a benign-appearing clinical course may be managed with continued imaging surveillance in selected cases. These considerations should be interpreted as supportive clinical context rather than definitive decision thresholds.

This study has several limitations. First, this was a retrospective single-center study with a small sample size and only nine malignant cases, which limited statistical power, increased the risk of overfitting in the multivariable model, and resulted in wide confidence intervals. Second, selection bias is inherent because only patients with NTM infection who underwent PCNB were included, representing a selected subgroup with clinically or radiologically indeterminate lesions. Case identification via diagnostic codes and radiology report keyword searches may have introduced additional ascertainment bias. Third, the cohort included patients biopsied in different clinical contexts, and detailed smoking history and COPD-related clinical data were not fully available, leaving potential residual confounding, particularly in the observed association between emphysema and malignancy. Finally, PET-CT was not included, which could provide additional discriminative value, and CT acquisition protocols varied across scanners. Despite these limitations, this study provides preliminary real-world data on biopsy-proven pulmonary lesions in patients with NTM infection, and the findings should be considered exploratory and require validation in larger prospective multicenter studies.

In conclusion, in patients with NTM infection who undergo PCNB for suspicious pulmonary lesions, lobulated irregular morphology and the presence of emphysema were associated with malignancy in this study. These findings may help refine clinical suspicion and support biopsy decision-making in selected cases, but they should be considered preliminary and require validation in larger multicenter studies before broader clinical application.

## Figures and Tables

**Table 1:** Clinical characteristics and pathologic results of PCNB.

			Group 1 (*n* = 22)	Group 2 (*n* = 16)
Clinical characteristics				
	Age (year, mean ± SD)		70.3 ± 7.4	67.4 ± 11.2
	Sex	Male	16 (72.7)	7 (43.8)
		Female	6 (27.3)	9 (56.3)
	Median time interval between diagnosis of NTM-PD and PCNB (days, median [IQR])		551.5 [372.5–1021.0]	6.5 [4.0–8.8]
	NTM species			
		*Mycobacterium avium*	13 (59.1)	6 (37.5)
		*Mycobacterium intracellulare*	4 (18.2)	6 (37.5)
		*Mycobacterium kansasii*	0 (0.0)	1 (6.3)
		*Mycobacterium fortuitum complex*	2 (9.1)	1 (6.3)
		Unknown	3 (13.6)	2 (12.5)
	Underlying malignancy			
		Yes	8 (36.4)	2 (12.5)
		None	14 (63.6)	14 (87.5)
Pathologic results of PCNB				
	Malignancy		7 (31.8)	2 (12.5)
		Poorly differentiated malignancy	1 (4.5)	0 (0.0)
		Squamous cell carcinoma	4 (18.2)	1 (6.3)
		Adenocarcinoma	1 (4.5)	1 (6.3)
		Metastatic infiltrating urothelial carcinoma from urinary bladder	1 (4.5)	0 (0.0)
	Benign		15 (68.2)	14 (87.5)
		Chronic inflammation with necrosis	6 (27.3)	3 (18.8)
		Chronic inflammation	5 (22.7)	6 (37.5)
		Necrosis	0 (0.0)	5 (31.3)
		Anthracofibrosis	3 (13.6)	0 (0.0)
		Fungal ball, consistent with Aspergillus	1 (4.5)	0 (0.0)

SD standard deviation, NTM-PD nontuberculous mycobacterial pulmonary disease, PCNB percutaneous core needle biopsy, IQR interquartile range.

**Table 2:** Chest CT findings of PCNB.

Chest CT Findings			Group 1 (*n* = 22)	Group 2 (*n* = 16)
	Location			
		Right upper lobe	6 (27.3)	1 (6.3)
		Right middle lobe	1 (4.5)	2 (12.5)
		Right lower lobe	6 (27.3)	7 (43.8)
		Left upper lobe	4 (18.2)	3 (18.8)
		Left lower lobe	5 (22.7)	3 (18.8)
	Morphologic appearance			
		Lobulated irregular nodule	11 (50.0)	8 (50.0)
		Smooth round nodule	5 (22.7)	3 (18.8)
		Patchy consolidation	4 (18.2)	5 (31.3)
		Subpleural thickening	2 (9.1)	0 (0.0)
	Size (mm, mean ± SD)		34.9 ± 17.8	33.6 ± 13.9
	Cavity		5 (22.7)	4 (25.0)
	Necrosis		7 (31.8)	8 (50.0)
	Associated multiple nodules			
		Yes	16 (72.7)	14 (87.5)
		No	6 (27.3)	2 (12.5)
	Underlying two typical forms of NTM-PD			
		Nodular bronchiectatic	12 (54.5)	5 (31.3)
		Upper lobe fibrocavitary	3 (13.6)	0 (0.0)
		None	7 (31.8)	11 (68.7)
	Interval change of PCNB lesion on previous follow-up CT			
		Newly appeared	5 (22.7)	3 (18.8)
		Gradual growth	14 (63.6)	6 (37.5)
		Fluctuation	2 (9.1)	0 (0.0)
		No change	0 (0.0)	3 (18.8)
		N/A	1 (4.5)	4 (25.0)
	Interval change of other lesions			
		Yes	8 (36.4)	6 (37.5)
		No	12 (54.5)	5 (31.3)
		N/A	2 (9.1)	5 (31.3)
	Emphysema		11 (50.0)	4 (25.0)
	Lymphadenopathy		6 (27.3)	5 (31.3)
	Pleural effusion		1 (4.5)	3 (18.8)

SD standard deviation, NTM-PD nontuberculous mycobacterial pulmonary disease, PCNB percutaneous core needle biopsy, N/A not applicable.

**Table 3:** Comparison of clinical and radiologic characteristics between benign and malignant lesions and logistic regression analysis.

		Benign (*n* = 29)	Malignant (*n* = 9)	*p*-Value	Univariable Logistic Regression		Multivariable Logistic Regression	
					*p*-Value	Odds Ratio	*p*-Value	Odds Ratio
Age (year, mean ± SD)		67.3 ± 9.1	74.7 ± 8.6	0.045	0.049	1.106 [1.000–1.222]		
Sex				0.061	0.074	7.467 [0.825–67.573]		
	Male	15 (51.7)	8 (88.9)					
	Female	14 (48.3)	1 (11.1)					
Location				0.321	0.53			
	Right upper lobe	6 (20.7)	1 (11.1)					
	Right middle lobe	3 (10.3)	0					
	Right lower lobe	10 (34.5)	3 (33.3)					
	Left upper lobe	6 (20.7)	1 (11.1)					
	Left lower lobe	4 (13.8)	4 (44.4)					
Size (mm, mean ± SD)		33.5 ± 17.3	39.7 ± 14.8	0.306	0.329	1.022 [0.978–1.069]		
Cavity or necrosis		19 (65.5)	4 (44.4)	0.436	0.265	0.421 [0.092–1.928]		
Shape *				0.057	0.023	13.091 [1.436–119.338]	0.023	19.856 [1.516–260.089]
	Lobulated irregular nodule	11 (37.9)	8 (88.9)					
	Smooth round nodule	7 (24.1)	1 (11.1)					
	Patchy consolidation	9 (31.0)	0					
	Subpleural thickening	2 (6.9)	0					
Associated multiple nodules				0.071	0.061	0.200 [0.037–1.080]		
	Yes	25 (86.2)	5 (55.6)					
	No	4 (13.8)	4 (44.4)					
Underlying two typical forms of NTM-PD				0.717	0.722			
	Nodular bronchiectatic	14 (48.3)	3 (33.3)					
	Upper lobe fibrocavitary	2 (6.9)	1 (11.1)					
	None	13 (44.8)	5 (55.6)					
Interval change of PCNB lesion on previous follow-up CT				0.356	0.986	0.714 [0.128–3.995]		
	Newly appeared	5 (17.2)	3 (33.3)					
	Gradual growth	14 (48.3)	6 (66.7)					
	Fluctuation	2 (6.9)	0					
	No change	3 (10.3)	0					
	N/A	5 (17.2)	0					
Interval change of other lesions				0.094	0.09	7.091 [0.737–68.236]		
	Yes	13 (44.8)	1 (11.1)					
	No	11 (37.9)	6 (66.7)					
	N/A	5 (17.2)	2 (22.2)					
Emphysema		7 (24.1)	8 (88.9)	<0.001	0.005	25.143 [2.660–237.624]	0.005	35.531 [2.857–441.824]
Lymphadenopathy		7 (24.1)	4 (44.4)	0.241	0.248	2.514 [0.525–12.036]		
Pleural effusion		2 (6.9)	2 (22.2)	0.233	0.214	3.857 [0.459–32.424]		

SD standard deviation, NTM-PD nontuberculous mycobacterial pulmonary disease, PCNB percutaneous core needle biopsy, N/A not applicable; * For logistic regression analysis, lesion shape was dichotomized as lobulated irregular nodule versus other morphologic patterns.

## Data Availability

The data presented in this study are available on request from the corresponding author due to ethical restrictions; access requires approval from the institutional ethics committee.

## Supplementary Materials

The following supporting information can be downloaded at: https://www.mdpi.com/article/10.3390/diagnostics16091321/s1, Table S1: Interobserver agreement between two readers for chest CT findings.
