# Supplementary material for: Differentiation of Lung Malignancy from Benign Lesions in Patients with Nontuberculous Mycobacterial Infection: A Retrospective Analysis of Biopsy-Proven Cases
Source: Diagnostics (Basel). 2026 Apr 28;16(9):1321. doi: 10.3390/diagnostics16091321 (PMC13163359; doi:10.3390/diagnostics16091321)
Supplement: Supplementary file 1 [file diagnostics-16-01321-s001.zip › diagnostics-4210585-supplementary.pdf]

Supplementary Table S1. Interobserver agreement between two readers for chest CT findings

	Reader 1	Reader 2	Interobserver agreement (95% CI)
Cavity or necrosis	23 (60.5)	21 (55.3)	0.892 (0.748–1.000)
Shape			0.919 (0.811–1.000)
Lobulated irregular nodule	19 (50.0)	18 (47.4)	
Smooth round nodule	8 (21.1)	8 (21.1)	
Patchy consolidation	9 (23.7)	11 (28.9)	
Subpleural thickening	2 (5.3)	1 (2.6)	
Associated multiple nodules	30 (78.9)	30 (78.9)	0.842 (0.629–1.000)
Underlying two typical forms of NTM-PD			0.907 (0.782–1.000)
Nodular bronchiectatic	17 (44.7)	15 (39.5)	
Upper lobe fibrocavitary	3 (7.9)	3 (7.9)	
None	18 (47.4)	20 (52.6)	
Interval change of PCNB lesion on previous follow-up CT			0.707 (0.515–0.898)
Newly appeared	8 (21.1)	8 (21.1)	
Gradual growth	20 (52.6)	22 (57.9)	
Fluctuation	2 (5.3)	1 (2.6)	
No change	3 (7.9)	2 (5.3)	
N/A	5 (13.2)	5 (13.2)	
Interval change of other lesions			0.750 (0.562–0.938)
Yes	15 (39.5)	13 (34.2)	
No	16 (42.1)	18 (47.4)	
N/A	7 (18.4)	7 (18.4)	
Emphysema	15 (39.5)	13 (34.2)	0.887 (0.736–1.000)
Lymphadenopathy	11 (28.9)	12 (31.6)	0.938 (0.817–1.000)
Pleural effusion	4 (10.5)	4 (10.5)	1.000 (1.000–1.000)

NTM-PD nontuberculous mycobacterial pulmonary disease, *PCNB* percutaneous core needle biopsy, *N/A* not applicable
